# Developing a genomic-based strategy to confirm microbial identity in bio-inputs containing multiple strains: an easy, fast, and low-cost multiplex PCR applied to inoculants carrying soybean *Bradyrhizobium*

**DOI:** 10.1007/s42770-024-01441-8

**Published:** 2024-07-12

**Authors:** Amanda Alves de Paiva Rolla-Santos, Leonardo Araujo Terra, Renan Augusto Ribeiro, Marco Antonio Nogueira, Mariangela Hungria

**Affiliations:** 1 CNPq, Ed. Telemundi II, SAUS, Quadra 01 Lotes 1 E 6, CEP, Brasília, Federal District Brazil; 2 Embrapa Soja, Soil Biotechnology Laboratory, C.P. 4006, 86.085-981, Londrina, Paraná Brazil

**Keywords:** *Bradyrhizobium*, Multiplex PCR, Inoculants, Inoculant identification, Bio-inputs

## Abstract

Brazil stands out in research, industrial development, and farmers' use of microbial inoculants, with an emphasis on getting benefits from the biological nitrogen fixation process with the soybean crop. Nowadays, about 140 million doses of inoculants are commercialized annually for the soybean in the country, and strain identification is achieved by rep-PCR, an effective but time-consuming method. Aiming to develop an easy, low-cost, and low-time-consuming method, we used a complete genome-based approach based on the unequivocal identification of unique genes present in the genomes of each of the four *Bradyrhizobium* strains used in commercial inoculants: *Bradyrhizobium elkanii* strains SEMIA 587 and SEMIA 5019, *Bradyrhizobium japonicum* SEMIA 5079, and *Bradyrhizobium diazoefficiens* SEMIA 5080. The unique pairs of primers able to amplify genomic regions of different sizes allowed the identification of the four strains in a simple multiplex polymerase chain reaction (PCR). Validation was confirmed by using single colonies, multiple cultures, and commercial inoculants. The number of labor hours of a technician was 3.08 times higher, and the final cost was 3.25 times higher in the rep-PCR than in the multiplex PCR. Most importantly, the results for multiplex PCR were obtained on the same day, in contrast with 15 days in the traditional methodology. The genomic approach developed can be easily applied to a variety of microbial inoculants worldwide, in addition to studies of ecology and evaluation of the competitiveness of the strains.

**Graphical Abstract:**

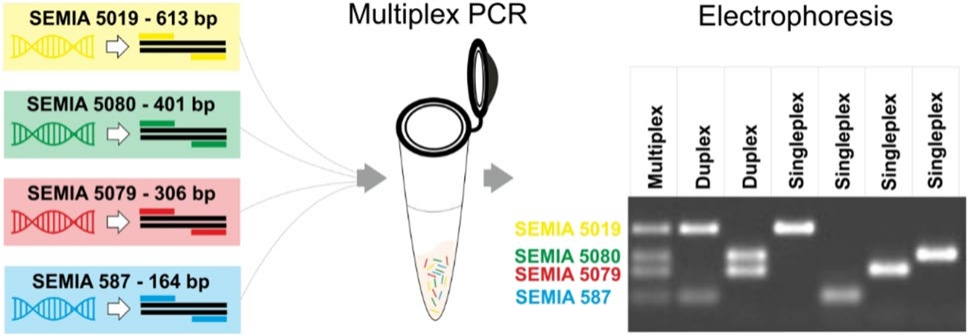

**Supplementary Information:**

The online version contains supplementary material available at 10.1007/s42770-024-01441-8.

## Introduction

Brazil has the most successful case of use of microbial inoculants benefiting agriculture, represented by rhizobial strains symbionts of the soybean (*Glycine max* (L.) Merr.) crop [1–5]. The success is based on more than seven decades of research, industrial development, and extension activities toward maximizing the contribution of the biological nitrogen fixation process [5]. As the soybean is an exotic plant in Brazil, the soils are originally void of compatible soybean bradyrhizobia [6]. Therefore, foreign strains were brought to the country mainly in the 1950s-1960s and searched for elite rhizobia compatible with the soybean genotypes cropped at that time in the southern states and also adapted to the local edaphoclimatic conditions [1, 2]. Two strains identified and released for the farmers in 1966 and 1968, *Bradyrhizobium elkanii* SEMIA 587 and SEMIA 5019, respectively, are so effective in fixing nitrogen that are used in commercial inoculants till today [5].

Strain selection programs have never stopped in Brazil. The expansion to the Cerrado region in the 1970s, today the main soybean cropping area in the country, demanded a new and innovative approach. The strategy consisted of searching variant strains from previous studies, but more efficient in fixing nitrogen and more competitive, after passing an adaptation period in the stressing conditions of the Cerrado´s soils [2]. This strategy resulted in the release, in 1992, of two outstanding strains, *Bradyrhizobium japonicum* SEMIA 5079 (= CPAC 15) and *Bradyrhizobium diazoefficiens* SEMIA 5080 (= CPAC 7) [7].

Based on several sources (e.g. https://www.anpii.org.br/) we estimate the commercial inoculant market in Brazil at about 170 million doses in 2024, with over 80% represented by bradyrhizobia for the soybean crop. For these inoculants, the Brazilian legislation requires a concentration of at least 10^9^ cells g^−1^ or mL^−1^ of the product, showing no contaminants at the 10^–5^ dilution, and containing exclusively the reported strains [8]. Only strains authorized by the legislation may compose the inoculants that, for the soybean, are represented by four strains: SEMIAs 587, 5019, 5079, and 5080 [9]. Ensuring that the inoculants carry one or more out of the four strains authorized for use in commercial inoculants is critical. The official method for strain identification is the rep-PCR [8, 10], based on the amplification of repetitive intergenic regions of the DNA [11–13]. Despite being robust, reliable, and effective in identifying rhizobial strains [10, 14, 15], the method is time- and labor-consuming, taking days to complete all steps. Therefore, the method does not apply to quality control in the industry, where decisions must be made daily and on a large scale.

We used a complete genome-based approach to develop an easy, low-cost, and low-time-consuming method. The genomes of the four soybean bradyrhizobia strains were analyzed in detail to search for unique genomic regions with different sizes. We describe the strategy, development, and validation of the method that allowed the fast and unequivocal identification of the four soybean bradyrhizobia strains in a multiplex PCR reaction. The approach for the method development can be easily applied to a variety of microbial inoculants commercialized worldwide, highly contributing to the quality control of bio-inputs. It can also be applied to other important studies, such as ecology and evaluation of the competitiveness of strains.

## Material and methods

### Strains, growth conditions and DNA extraction

The strains of this study, composed of *B. elkanii* SEMIA 587 (= CNPSo 14) and SEMIA 5019 (= CNPSo 09), *B. diazoefficiens* SEMIA 5080 (= CNPSo 06, = CPAC 7), and *B. japonicum* SEMIA 5079 (= CNPSo 07, = CPAC 15), as well as of *Azospirillum brasilense* strain CNPSo 2083 (= Ab-V5), used as negative control in the multiplex PCR reaction, were obtained at the “Diazotrophic and Plant Growth Promoting Bacteria Culture Collection of Embrapa Soja” (WFCC Collection No. 1213, WDCM Collection No. 1054), Londrina, Paraná, Brazil.

Bacterial strains SEMIA 587, 5019, 5079, and 5080 were grown in a modified-yeast-extract-mannitol (YM) medium [16] and incubated at 28 °C with constant shaking until they reached the exponential growth phase (OD_600_ 0.5 to 0.7). Subsequently, 1.5 mL of each bacterial culture was subjected to DNA extraction using the DNeasy Blood and Tissue kit (Qiagen®), following the manufacturer's instructions. For the four different commercial liquid inoculants tested, 1.5 mL of each inoculant was used for DNA extraction also with the DNeasy Blood and Tissue kit (Qiagen®. After extraction, all DNA samples were quantified using a Qubit fluorometer (Invitrogen).

To check the technique's detection limit, mixtures of bacterial cultures in different proportions (10:90 to 40:60), represented by the combinations of SEMIA 587 + SEMIA 5019 and SEMIA 5079 + SEMIA 5080, which compose more than 99% of the commercial market of soybean inoculants in Brazil were also used. The cultivation was carried out in modified-YM medium, the OD600 was adjusted to 0.4 to 0.5, and DNA was extracted.

### Selection of candidate genes

A total of 267 *Bradyrhizobium* genomes of 68 different species of *Bradyrhizobium* were retrieved from the NCBI website (www.ncbi.nih.gov) and used in this study. Among these, the complete genome of *B. japonicum* SEMIA 5079 recovered from GenBank (CP007569.1), of *B. diazoefficiens* SEMIA 5080 (CP139636.1), and two incomplete genomes of *B. elkanii* SEMIA 587 and 5019 (unpublished data from our laboratory) derivatives were included in the study.

All 267 genomes were automatically annotated using the Prokka version 1.14.6 program [17]. Then, the multifast gene files of the 267 genomes were concatenated and used to build the local BLAST database. The multifast gene sequences of SEMIA strains 587, 5019, 5079, and 5080 were used as a query for a BLASTn search with default parameters [18]. Genes with a maximum of ten hits with the nearby strains in the BLAST sequence analysis results were selected as candidate genes (Online Resource; Table S1). The multifast file was built using the SeqKit tool version 2.3.0 [19]. The online BLAST-NCBI Whole-Genome Shotgun contigs (WGS) database for the taxon *Bradyrhizobiaceae* (taxid: 41294) was used using default parameters to check the presence of other strains in the selected candidate genes. More specifically, ten hits with SEMIA 5080, four with SEMIA 5079, two with SEMIA 587, and none with SEMIA 5019. In this study, all candidate genes selected were hypothetical genes.

### Primer design

The specific primers for *Bradyrhizobium* strains SEMIAs 587, 5019, 5079, and 5080 were designed using the online tool Primer3Plus (https://www.primer3plus.com/index.html) to assist in the best genome locations, and finished manually with the OLIGOEXPLORER 1.5 program (http://www.genelink.com). The main parameters for the design of the multiplex PCR primers were: size between 18 and 24 nt; CG content between 41.7% and 61.1%; primer annealing temperature between 59.2% and 60.5%; and minimum difference between the 95 bp amplicons.

Table 1 lists the specific primer sequences used in this study. All primers were synthesized by IDT (Integrated DNA Technologies, Newark, NJ).

**Table 1:** Primers identified for the four soybean *Bradyrhizobium* strains carried in commercial inoculants in Brazil for the use in the multiplex PCR reaction

Strain	Primer sequence (5’—> 3’)	bp	Amplicom size (bp)
*B. elkanii* SEMIA 587 (‒CNPSo 14)	GAAAGACTGGAACGAGGAGC	20	164
	GCGAGATAATCCGAAGATGCAC	22	
*B. japonicum* SEMIA 5079 (‒CNPSo 07)	GACGCCCAGCAACGAAATAC	20	306
	ACGCTCACACACTCTTGGTT	20	
*B. diazoefficiens* SEMIA 5080 (‒CNPSo 06)	GCACAAATCGGGAAATTATGAGGT	24	401
	GCTTTACTTTGTTGCTCAACGCG	23	
*B. elkanii* SEMIA 5019 (‒CNPSo 09)	CTGCCGTTGTGTTCTATTTCTTCG	24	613
	TCTATGTCGCCGTCGCTC	18	

### Optimization singleplex PCR

Initially, singleplex PCR reactions were performed to verify the specificity of the primers with the target strain. The PCR reactions of the protocol were optimized using the GoTaq® Flexi DNA Polymerase (Promega, Madson, WI), with the following conditions: 5 µL of 5X Green GoTaq® Flexi Buffer, 1.16 µL of 25 mM MgCl_2_ solution, 1.5 µL of 10 mM of each deoxyribonucleotide triphosphate (dATP, dCTP, dGTP, dTTP) (Invitrogen, Carlsbad), 0.18 µL of GoTaq® DNA Flexi DNA Polymerase (5U µL^−1^), 0.75 µL of each primer at 10 µM for SEMIAs 587, 5079 and 5080 and 0.75 µL at a concentration of 20 µM for SEMIA 5019, in addition to 1.2 µL of genomic DNA (40–80 ng µL^−1^, with DNA quantified using a Qubit fluorometer, Invitrogen) and sterile water recommended for molecular biology, totaling the final volume 25 μL.

### Multiplex PCR

To verify the detection limit of each strain, duplex PCR protocol was used, using bacterial liquid culture varying between 10 and 90% of the combinations SEMIAs 587 + 5019 and 5079 + 5080, with the following reactions occurring: GoTaq® Flexi DNA Polymerase (Promega, Madson, WI), and: 5 µL of 5X Green GoTaq® Flexi Buffer, 1.16 µL of 25 mM MgCl_2_ solution, 1.5 µL of 10 mM of each deoxyribonucleotide triphosphate (dATP, dCTP, dGTP, dTTP) (Invitrogen, Carlsbad), 0.18 µL of GoTaq® DNA Flexi DNA Polymerase (5U µL^−1^), 0.75 µL of each primer at 10 µM for SEMIA 587, 0.75 µL of each primer at 20 µM for SEMIA 5019, 0.75 µL of each primer at 10 µM for SEMIAs 5079, and 0.75 µL of each primer at 10 µM for SEMIA 5080, plus 1.2 µL of genomic DNA (40–80 ng µL^−1^) and sterile water recommended for molecular biology, totaling a final volume of 25 μL.

**Fig. 1:**
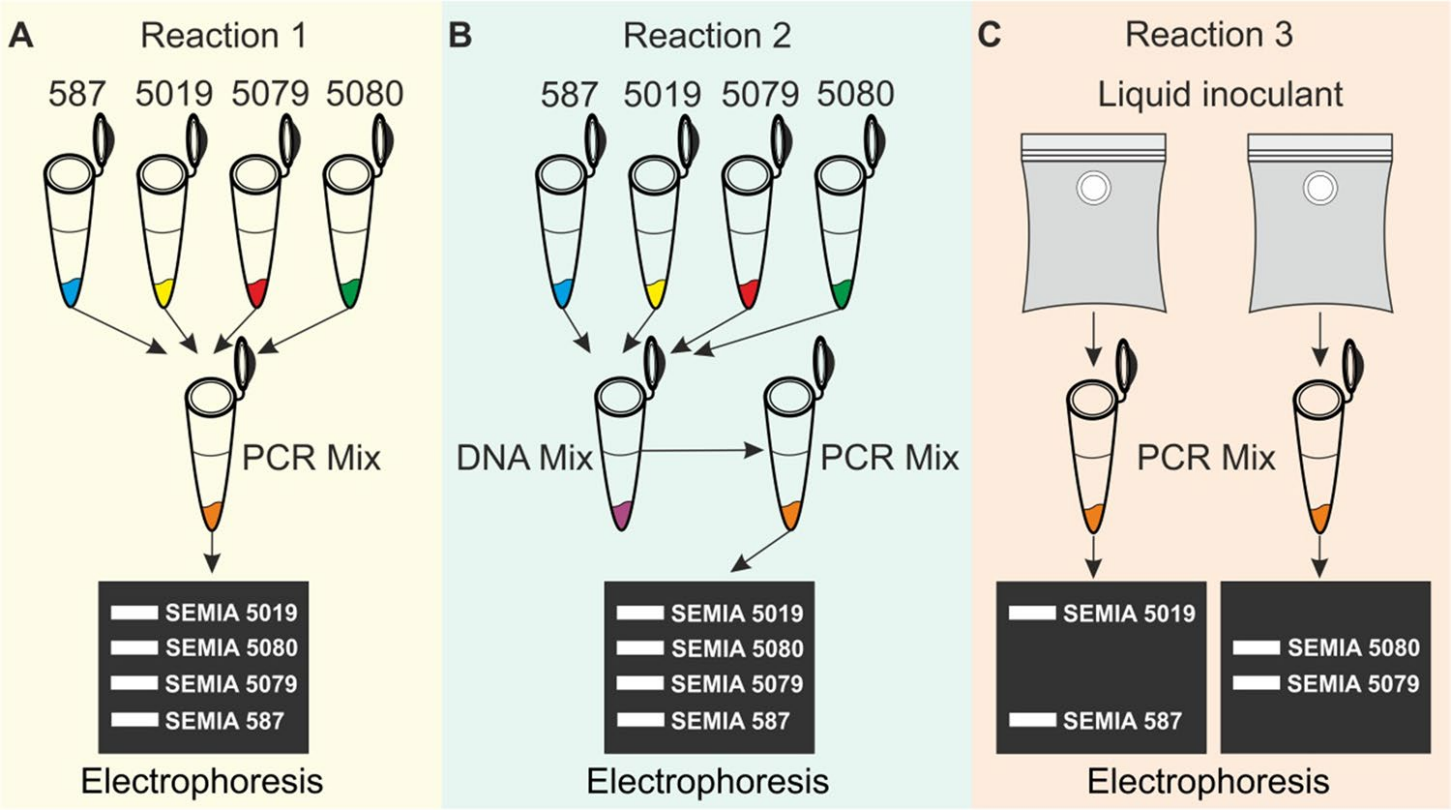
Different methods for preparing samples for multiplex PCR. **A**) The DNA of the strains are mixed directly in the tube with the PCR mix. **B**) DNAs of all four strains are mixed before the PCR mix. **C**) DNA is extracted directly from the liquid inoculant and added to the PCR mix

Before the multiplex PCR (quadruplex) step, samples were prepared in different ways to ensure amplification success. In multiplex PCR (quadruplex) reactions, four sets of primers referring to the four strains were used in the same reaction. Three different approaches were established: 1. The genomic DNA of each strain was added to the tube containing all components of the PCR reaction, with a volume of 1.2 µL for each strain (Fig. 1A); 2. The genomic DNAs of the four strains were mixed in a single tube, homogenized, and then an aliquot of 4.8 µL was taken from this mixture and added directly to the tube containing the components of the PCR reaction (Fig. 1B); 3. The volume of 1.2 µL of genomic DNA was extracted directly from the inoculant sample, supplemented with 3.6 µL of nuclease-free water, and added to the mixture containing all components of the PCR reaction (Fig. 1C).

The Multiplex PCR allows the simultaneous amplification of multiple target sequences in a single reaction using multiple primer pairs. For amplification of the four strains in a single tube, the PCR reaction was prepared following the conditions in Table 2.

**Table 2:** Reagents used in the multiplex PCR

Components	Final concentration	Final volume		
5X Green GoTaq® Flexi Buffer	1X	5 µL		
PCR Nucleotide Mix, 10 mM each	0.6 mM each dNTP	1.5 µL		
MgCl_2_ Solution_,_ 25 mM	1.16 mM	1.16 µL		
Primer Forward 10 µM – SEMIA 587 Primer Reverse 10 µM – SEMIA 587 Primer Forward 20 µM – SEMIA 5019 Primer Reverse 20 µM – SEMIA 5019 Primer Forward 10 µM – SEMIA 5079 Primer Reverse 10 µM – SEMIA 5079 Primer Forward 10 µM – SEMIA 5080 Primer Reverse 10 µM – SEMIA 5080	0.3 µM 0.3 µM 0.6 µM^a^ 0.6 µM 0.3 µM 0.3 µM 0.3 µM 0.3 µM	0.75 µL 0.75 µL 0.75 µL 0.75 µL 0.75 µL 0.75 µL 0.75 µL 0.75 µL		
GoTaq® Flexi DNA Polymerase (5U µL^−1^)	0.9 U	0.18 µL		
	‒	Reaction 1	Reaction 2	Reaction 3
Template DNA [40]-[80]ng µL^−1^		1.2 µL 587 1.2 µL 5019 1.2 µL 5079 1.2 µL 5080	4.8 µL mix	1.2 µL inoculant + 3.6 µL water
Sufficient quantity of water^b^	‒	6.36 µL		
Final reaction volume	‒	25 µL		

^a^ Double concentration of the primer pair for SEMIA 5019

^b^ Nucleases-free water

### Optimization of PCR conditions for singleplex and multiplex reactions

PCR amplification was performed in the ProFlex™ 3 × 32-well PCR System Thermocycler (Applied Biosystems™) under the following conditions: initial denaturation at 95 °C for 2 min, followed by 35 cycles of 95 °C for 45 s, 65 °C for 30 s, 72 °C for 1:30 min, final extension step at 72 °C for 7 min and final 4 °C. The PCR products (5 µL each sample) were separated on 1.5% agarose gels (10 × 5 cm size) in 1 × Tris–borate-EDTA (TBE) buffer, applying 60 V per 60 min, stained with ethidium bromide, and visualized on the L-PIX UV transilluminator (Loccus® do Brazil Ltda). A molecular weight marker of 1 kb plus DNA ladder (ThermoFisher Scientific, USA) was included in each gel.

## Results

The candidate genes for each strain were selected after the analysis of 267 *Bradyrhizobium* genomes and manual curation using the WGS database to create specific primers. The curation analysis indicated that the genes selected for *B. elkanii* strain SEMIA 587, *B. japonicum* strain SEMIA 5079, and *B. diazoefficiens* strain 5080 matched genetically close strains, while strain *B. elkanii* 5019 did not match any strain (Online Resource 1; Table S1). It is worth mentioning that the other strains presented in the supplementary material are not used as inoculants and, therefore, do not make our work unfeasible.

The first step of this study focused on identifying unique and exclusive genes of each of the four strains, using liquid cultures of single strains. One primer pair was designed for each of the strains SEMIA 587, 5019, and 5079, and two primer pairs were designed for strain SEMIA 5080. All designed primers were successful for the amplification. The most critical step in designing the primers for the multiplex PCR protocol was to design primers that presented similar annealing melting temperatures with all primers present in the same reaction and that did not have targets in regions present in the mixture of genomic DNA with the other strains. Furthermore, to avoid problems during amplification and to increase the efficiency of the primers, the formation of secondary structures between the same primers and the formation of dimers between different primers were verified.

The second step involved optimizing some components of the PCR reaction to ensure the amplification of all regions of the four strains in a single reaction in the multiplex PCR system, with the minimum difference of 95 bp to visualize distinct bands in the agarose gel. This step involved several adjustments in the concentrations of magnesium ions and also in the concentrations of the primers in the mixture, aiming at adjusting the amplification efficiency of the amplicons. Several tests were also necessary with different commercial DNA polymerase enzymes to select the most suitable enzyme that presented amplification of all bands in a single reaction. Furthermore, the in silico annealing temperature for the four primer pairs initially established in the range of 59.2 to 60.5% was subjected to several singleplex PCR reactions. After several tests, the annealing temperature of 65 ºC showed satisfactory amplification for all targets.

**Fig. 2:**
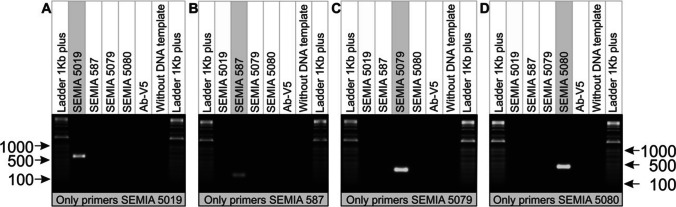
Assessment of primer specificity in the identification of four commercial strains of soybean *Bradyrhizobium* in a singleplex amplification containing all primer pairs of the four strains. Specific amplification in **A**) SEMIA 5019; **B**) SEMIA 587; **C**) SEMIA 5079; **D**) SEMIA 5080. *Azospirillum brasilense s*train Ab-V5 was used as a negative control

To evaluate the specificity of the primers, genomic DNA from each strain was used in separate multiplex PCR reactions containing all primer pairs from the four strains on each agarose gel (Fig. 1 A, B). Specific amplification was successful, showing only the expected fragment from the strain to its corresponding primer on each agarose gel, indicating that the selected primers were congruent in identifying their target strain (Fig. 2).

**Fig. 3:**
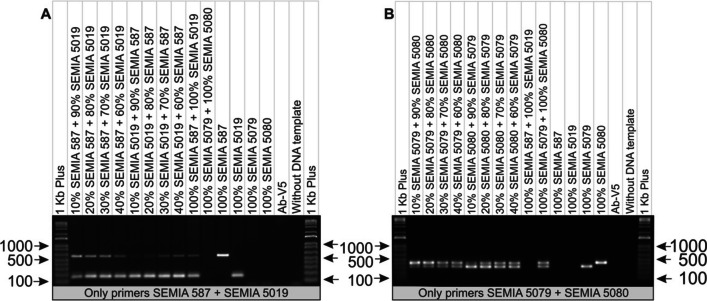
**A**) Limit of detection at different proportions (10 to 90%) for *Bradyrhizobium* spp. strains SEMIA 587 + SEMIA 5019 and controls in a multiplex reaction. **B**) Limit of detection at different proportions (10 to 90%) for strains SEMIA 5079 + SEMIA 5080 and controls in a multiplex reaction. *Azospirillum brasilense* strain Ab-V5 was used as a negative control

The third stage addressed the duplex reaction using different proportions of bacterial liquid culture for each strain ranging from 10 to 90% to evaluate the sensitivity of the protocol. This step involved the combination of two strains in a single reaction: a set of primers for the combination of SEMIA 587 + SEMIA 5019 and another set for the combination of SEMIA 5079 + SEMIA 5080 strains for simultaneous amplification of the DNA of these strains. The strains were identified by agarose gel electrophoresis analysis, which made it possible to distinguish the samples based on the size of the amplified fragments (Fig. 3). The results demonstrated well-defined and congruent bands, highlighting the high sensitivity and specificity of the primers in relation to their specific targets under the conditions established in the duplex reaction.

In the fourth and final step, after validating the specificity of the primers in relation to the target strains and determining the detection limit by varying the DNA concentrations of each strain, the multiplex PCR (quadruplex) method test was performed. This evaluation allowed the amplification of multiple DNA fragments of the four strains simultaneously.

**Fig. 4:**
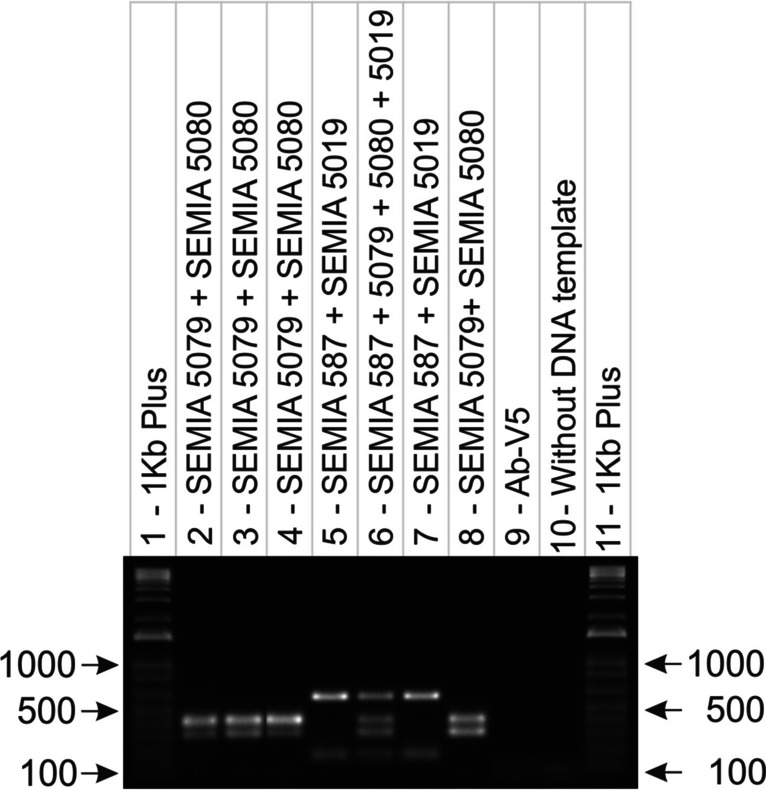
Detection by multiplex PCR reaction of the target strains of this work. Lanes: 1. Molecular marker 1 kb; 2—5. Different commercial inoculants for soybeans (SEMIA 587, 5019, 5079, and 5080); 6. Laboratory liquid culture containing four strains (SEMIA 587 + 5019 + 5079 + 5080); 7. Laboratory liquid culture containing two strains (SEMIA 587 + 5019); 8. Laboratory culture containing two strains (SEMIA 5079 + 5080); 9. Negative control; 10. Reagent without template DNA; 11. Molecular marker 1 kb

In our experiment, we combined the DNA of the four strains, SEMIA 587, SEMIA 5019, SEMIA 5079, and SEMIA 5080, and their respective specific primers in a single reaction, which allowed the distinct visualization of the four bands in the agarose gel (Fig. 4). Furthermore, to evaluate the effectiveness of the multiplex PCR methodology when applied directly to samples of commercial inoculants in liquid culture medium, our multiplex PCR protocol was able to accurately identify bacterial strains present in different commercial inoculants available on the market. Analysis of the results obtained confirmed the effectiveness and specificity of our method (Fig. 4).

To evaluate the volume added to the PCR mix of genomic DNA extracted directly from commercial inoculant samples, the volume of DNA was adjusted from 1.2 µL to 4.8 µL. This increase in volume resulted in poorly defined DNA bands in the electrophoresis gel, indicating excess DNA (data not shown). Therefore, we recommend strictly following the protocol described in Table 2.

## Discussion

The unequivocal identification of the strains carried in a bio-input is critical to guarantee that all the work involved in the selection of microbial elite germplasm, in the industrial development, and in the validation of the agronomic efficiency of the product will reach the farmers. Certainly, the outstanding contribution of biological nitrogen fixation to the soybean crop in Brazil [2–5, 20] has strong support in the guarantee that the inoculants will carry only the elite strains identified by the research and legally authorized [9, 10].

Nowadays, the official method in Brazil to identify commercial strains is the rep-PCR [8, 10], developed in the 1990s and based on the amplification of conserved and repetitive intergenic regions of the DNA [11–13]. Since 1998 [21], our research group has published dozens of studies applying the rep-PCR method, including the profiles of all rhizobial strains authorized for the production of commercial inoculants in Brazil for leguminous grain, forage, green manure, and trees [14, 15]. However, the method is time- and labor-consuming and not applicable to making fast decisions in the industry, which today commercializes about 180 million doses annually of several bacterial strains for both legumes and non-legumes, with estimates of about 140 million of *Bradyrhizobium* for the soybean crop.

In contrast to the classical PCR system, where specific oligonucleotides are used to amplify a single DNA target and visualize it on an agarose gel, the multiplex PCR method allows for the simultaneous amplification of multiple target DNA fragments [22], identifying several organisms in a single reaction. This technique has already been broadly used to authenticate species in various food matrices, such as meat, fish, and dairy products [22].

In this context, the protocol presented in this study stands out for its robustness, cost–benefit, and reliability, dedicated to the identification of the strains of *B. elkanii* SEMIA 587 and SEMIA 5019, *B. diazoefficiens* SEMIA 5080, and *B. japonicum* SEMIA 5079. According to Brazilian legislation, all methods used for quality control of bio-inputs must be publicly available so that they can be broadly validated and freely used. Our study developed a reliable and easy protocol to confirm the identity of the four soybean-*Bradyrhizobium* strains used in commercial inoculants in Brazil. It is important to comment that these strains are also used in inoculants in other countries in South America, such as Paraguay and Bolivia. Although strains SEMIA 5079, SEMIA 5080, and SEMIA 587 matched very few other closely genetically related strains (Online Resource; Table S1), this does not compromise the viability of the multiplex PCR protocol, since such strains are not used in commercial inoculants in Brazil.

Our multiplex PCR protocol allowed the simultaneous amplification of the four target strains of this study in a single reaction, reducing labor time and cost, and offering a significant advantage over the conventional method of rep-PCR. The labor time and cost of the BOX-PCR and the multiplex PCR were compared. When analyzing soybean Brazilian commercial inoculants, all carrying two strains, eight colonies are used in the BOX-PCR analysis to confirm the presence of two strains [10], while in the multiplex PCR, the DNA can be extracted straight from the inoculant. Considering the materials and reagents used, the costs for the BOX-PCR were 5.5 times higher than the multiplex PCR, and 3.08 times higher in the number of labor hours of a technician, resulting in a final cost of 3.25 times higher for the BOX-PCR. Most importantly, the results were obtained on the same day in the multiplex PCR, in contrast with 15 days in the traditional methodology (Online Resource; Table S2; Figure S1).

This technique proved to be efficient with DNA isolated from pure strains from culture collections, and also with DNA isolated directly from commercial liquid inoculants. The standardization of our multiplex PCR protocol required many repetitions with different brands of the DNA polymerase enzyme, in addition to adjustments in magnesium ion concentrations, primer concentrations, and annealing temperature. To ensure the reliability of the multiplex PCR method, the protocol was tested under the same standardized conditions by different analysts from our laboratory, reaching the same results and consolidating the precision of the process. Noteworthy is that the same protocol steps can guide the development of protocols to identify a variety of unique strains used in commercial inoculants worldwide.

All economic analyses indicate that the rate of use of biological products in agriculture will be far greater than that of chemical products. An analysis of the global bio-inputs market was estimated at US$10.6 billion in 2021, with a predicted compound annual growth rate (CAGR) of 11.9% over the next five years to hit US$ 18.5 billion by 2026 [23]. Rates of adoption in Brazil are even higher than in other countries, with an expected increase of 17% in 2024 [24]. Therefore, fast, low-cost, and reliable protocols to guarantee the quality of the bio-inputs reaching the market, such as the one reported in this study, are key to guaranteeing more sustainable agricultural systems.

## Conclusion

The development and standardization of our protocol to identify the four *Bradyrhizobium* strains carried in commercial soybean inoculants in Brazil highlight the method's practical, fast, and low-cost use and ensure quality control regarding the identity of the elite strains. To guarantee that the strains identified by the research as the most effective is key to achieving the remarkable benefits of the biological nitrogen fixation process with the crop. It is also important that the method is freely available to the public and private sectors so that it can ensure the quality of the bio-inputs in scientific studies, in the industry, and in private and public laboratories. Noteworthy, the same protocol can be adapted to other strains used in any bio-input worldwide.

## Supplementary Information

Below is the link to the electronic supplementary material.

Supplementary file1 (XLSX 13 KB)

Supplementary file2 (DOCX 3.68 MB)

## Data Availability

All data and materials cited in the manuscript are freely available to the scientific community.
