# Supplementary material for: Developing a genomic-based strategy to confirm microbial identity in bio-inputs containing multiple strains: an easy, fast, and low-cost multiplex PCR applied to inoculants carrying soybean Bradyrhizobium
Source: Braz J Microbiol. 2024 Jul 12;55(3):2869–77. doi: 10.1007/s42770-024-01441-8 (PMC11405733; doi:10.1007/s42770-024-01441-8)
Supplement: Supplementary file 2 — Supplementary file2 (DOCX 3.68 MB) [file 42770_2024_1441_MOESM2_ESM.docx]

**Supplementary Table S2.** Comparison of cost and time in the analysis of one inoculant, considering eight colonies in the BOX-PCR or the inoculant with the multiplex PCR presented in this study. Costs estimated in Brazilian money (Real), and for the transformation to dollar the value in June of 2024 is of 1 dollar= 5.3 reais.

Comparison of Cost of BOX-PCR and Multiplex PCR

|  | **Cost of BOX-PCR** | | **Cost of Multiplex PCR** |
| --- | --- | --- | --- |
| **Material/Reagent** | Price R$ (to 1 sample) | Price R$ (8 colonies) | Price R$ (1 sample) |
| Sample buffer | 0.30 | 2.40 | **-** |
| Culture medium | 0.20 | 1.60 | **-** |
| Tubes | 1.20 | 9.60 | 1.20 |
| Tips | 0.70 | 5.60 | 0.70 |
| 1 kb | 1.98 | 1.98 | 1.98 |
| DNTPs | 0.20 | 1.60 | 0.20 |
| Taq Polymerase | 1.75 | 14.00 | 1.05 |
| Agarose | 30.00 | 30.00 | 12.00 |
| DNA extraction kit | 32.00 | 256.00 | 32.00 |
| Ethidium bromide | 2.60 | 2.60 | 2.60 |
| Boric acid/Tris-HCl | 5.00 | 5.00 | 5.00 |
| EDTA | 3.00 | 3.00 | 3.00 |
| RNAse | 0.64 | 5.12 | 0.64 |
| Primer | 0.35 | 2.80 | 2.80 |
| Labor of a technician (R$ 123,00/h) | 2,640.00 | 2,460.00 | 799.50 |
| **Total Cost** |  | **2,797.30** | **860.80** |
| **Cost considering only thematerial/ reagents** |  | **337.30** | **61.3** |

Time (Labour)

| **Methodology** | **BOX** | **Multiplex** |
| --- | --- | --- |
| Dilution and plating | 3 h | - |
| Growth* | 7 days | - |
| Selection and growth of colonies* | 5 days | - |
| DNA Extraction | 2 h | 2h |
| PCR | 7 h | 2h30mim |
| Electrophoresis | 8 h | 2h |
| Number of hours per 10 samples** | 20h (1200 min) | 6h30 min (390 min) |

* Not considered in the cost.

**Only the hours worked by the laboratory technician were considered.

Comparison in times of cost and days to obtain the results

| **Comparison of the two methodologies** | **BOX** | **Multiplex** |
| --- | --- | --- |
| Cost of reagents | 5.5x | 1x |
| Labor of a technician (R$ 123,00/h) | 3.08x | 1x |
| Total cost | 3.25x | 1x |
| Time to obtain the results | 15 days* | 1 day |

* Working a maximum of eight hours a day.

**Supplementary Figure S1.** Comparison of liquid detection methods: Multiplex PCR vs. BOX-PCR. Costs are in Brazilian money, “reais” (R$), in 06/2024 at the approximate rate of 5 R$ = 1 US$.
